# Spectral compression of single-photon-level laser pulse

**DOI:** 10.1038/srep43494

**Published:** 2017-02-27

**Authors:** Yuanhua Li, Tong Xiang, Yiyou Nie, Minghuang Sang, Xianfeng Chen

**Affiliations:** 1 State Key Laboratory of Advanced Optical Communication Systems and Networks, Department of Physics and Astronomy, Shanghai Jiao Tong University, Shanghai 200240, China; 2 Key Laboratory for Laser plasma (Ministry of Education), Collaborative Innovation Center of IFSA (CICIFSA), Shanghai Jiao Tong University, Shanghai 200240, China; 3 Department of Physics, Jiangxi Normal University, Nanchang 330022, China

## Abstract

We experimentally demonstrate that the bandwidth of single photons laser pulse is compressed by a factor of 58 in a periodically poled lithium niobate (PPLN) waveguide chip. A positively chirped single photons laser pulse and a negatively chirped classical laser pulse are employed to produce a narrowband single photon pulse with new frequency through sum-frequency generation. In our experiment, the frequency and bandwidth of single photons at 1550 nm are simultaneously converted. Our results mark a critical step towards the realization of coherent photonic interface between quantum communication at 1550 nm and quantum memory in the near-visible window.

Low loss transmission is an intrinsic and unique property for single photons at 1550 nm in optical fiber^1^,^2^,^3^. In quantum networks over optical fiber, single photons at 1550 nm are used for virtually all quantum information tasks, such as quantum metrology^4^, quantum computation^5^ and quantum cryptography^6^. Spontaneous parametric down-conversion (SPDC) sources are readily available for the production of entangled photon pairs at 1550 nm, and typically yield spectral bandwidths of 300 GHz^7^. Nevertheless, the narrowband photons in the near-visible wavelength possess the most efficient quantum memories and an ability of being easily detected by a silicon avalanche photodiode (APD). Therefore, it is highly expected that a coherent photonic interface is necessary which is capable of spectrum compressing and frequency conversing in the telecom band simultaneously.

Numerous schemes for spectral compression of broadband classical light have been demonstrated by using various compressor units and second-order nonlinear crystals^8^,^9^,^10^,^11^,^12^,^13^,^14^,^15^,^16^,^17^,^18^,^19^,^20^,^21^,^22^,^23^. Nonlinear frequency conversion could be used to generate the long narrowband pulse light^24^,^25^,^26^,^27^,^28^,^29^, such as sum frequency generation (SFG) in periodically poled lithium niobate (PPLN) waveguide^30^,^31^,^32^. Recently, a theoretical scheme has been proposed for spectral compression and frequency conversion of quantum light pulse^33^. The related experiment has been demonstrated that the spectrum of single photons was compressed by a factor of 40 in a β-barium-borate (BBO) crystal^34^. A negatively chirped intense laser pulse centred at 787.62 nm and a positively chirped single photon pulse centred at 811.11 nm were used to create a third single photon pulse centred around 399.7 nm through nonlinear optical process of SFG. The single photons were generated by spontaneous parametric down-conversion. An optical fiber and a pair of diffraction gratings were used to produce positively and negatively chirped pulses, respectively. Furthermore, the setup was for the most part a free-space optics one. Similar results have been achieved through temporal gating^35^ and room-temperature diamond quantum memory^36^. In the proposed methods of refs 34, 35, 36, spectrum compressing and frequency conversing technologies are not in 1550-nm telecommunications band, which is not appropriate for conventional quantum communication using all-optical-fiber networks. It is well known that single photons coherent state at 1550 nm is important in the development of future quantum communication tasks such as quantum key distribution^3^. To the best of our knowledge, spectral compression of single photons coherent state at 1550 nm has not been experimentally demonstrated yet.

In this Letter, we exploit a SFG process with a negatively chirped classical laser pulse and a positively chirped single photons laser pulse, which the bandwidth of the positively chirped single photons laser pulse is compressed in a PPLN waveguide chip—from 800 GHz to 13.7 GHz—which is approaching the bandwidth regime of some quantum memories^37^,^38^. In the same time, the 1550-nm telecom-band photons are flexibly converted into the near infrared window.

## Theoretical Description

In our experiment, spectral compressed single photons coherent pulse is generated by SFG between a negatively chirped classical laser pulse and a positively chirped single photons laser pulse. The negatively chirped classical laser pulse of frequency with *ν*_0,*P*_ increases in time *t*_1_, i.e., *ν*_0,*P*_(*t*_1_) = *ν*_0,*P*_ + *πt*_1_/*A*, and the positively chirped single photons pulse frequency with *ν*_0,*Q*_ decreases linearly in time *t*_2_, *ν*_0,*Q*_(*t*_2_) = *ν*_0,*Q*_ − *πt*_2_/*A*. Here *A* denotes the chirp rate, *ν*_0,*P*_ and *ν*_0,*Q*_ are the centre frequency of these two sources, and Δ*t* = *t*_1_ − *t*_2_ is a relative time delay between the negatively chirped classical laser pulse and positively chirped single photons laser pulse. When a positively chirped single photons laser pulse and a negatively chirped classical laser pulse arrive at the PPLN waveguide chip simultaneously, the frequency of *ν*_0,*Q*_(*t*_2_) would meet the frequency of *ν*_0,*P*_(*t*_1_) with the relative time delay Δ*t*, and SFG happen between two pluses, and the created single photon pulse with a narrow frequency bandwidth can be described *ν*_0,*SFG*_(Δ*t*) = *ν*_0,*P*_ + *ν*_0,*Q*_ + *π*Δ*t*/*A. ν*_0,*Q*_ and *ν*_0,*P*_ are equal from two replicas of the laser pulse source as the positively chirped single photons laser pulse and the negatively chirped classical laser pulse. The expected (TH) intensity bandwidth (FWHM) of the up-converted single photon is ref. 34


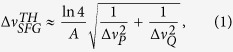


where Δ*ν*_*P*_ and Δ*ν*_*Q*_ are the FWHM bandwidths used in the waveguide. The bandwidth Δ*ν* is limited by group velocity dispersion, and decreases linearly with the length of the waveguide, i.e., 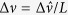, where 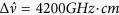 is the spectral acceptance of the waveguide. When the full FWHM bandwidths of negatively and positively chirped photons are used in the waveguide, the maximum SFG efficiency is guaranteed.

## Experimental Setup

**Figure 1:**
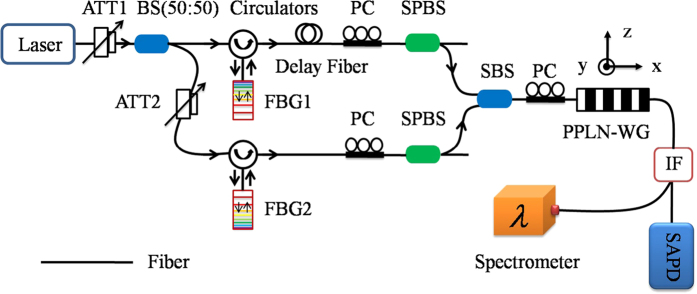
Experiment set-up. ATT, variable optical attenuator; BS, polarization maintaining beam splitter (50:50); FBG, fiber Bragg grating; Circulators, optical fiber circulators; Delay Fiber, optical adjustable delay fiber; PC, polarization controller; SPBS, single mode polarization beam splitter (single mode to polarization maintaining); SBS, single mode beam splitter (single mode to polarization maintaining); PPLN-WG, periodically poled lithium niobate waveguide chip; IF, interference filter; SAPD, Silicon APD.

The experimental setup is shown in Fig. 1. Two variable optical attenuators (ATT1 and ATT2) are used to create single photons pulse and control the energy of negatively chirped classical laser pulse for this experiment. The attenuation can also be realized by a polarization controller and a single mode polarization beam splitter. In our experiment, a mode-locked optical fiber laser pulse source has 500-fs duration, about 6.4 nm spectral FWHM bandwidth, 1551.54 nm centre wavelength at a 59.98 MHz repetition rate, and 45.2 mw average power.

Firstly, the laser pulse source is divided into two replicas using a 50:50 polarization maintaining beam splitter (BS), and then one of the two replicas of laser pulse is sent to a broadband fiber Bragg grating 1 (FBG1), and at the same time, the other laser pulse is coupled into the FBG2. The parameters of FBG1 and FBG2 are the exactly same (1547 nm centre wavelength, 39 nm FWHM bandwidth, and 5 nm/cm chirp rate). As ones know, FBG can be used for up-chirping and down-chirping, depending on the choice of the side from which the laser pulse is reflected. Thus, the two different chirp laser pulses after FBG1 and FBG2 are the same but with the opposite sign. It implies that the positively and negatively chirped pulses have equal and opposite chirp, ±*A*. A positively chirped laser pulse is generated through FBG2 to introduce a linear chirp by group velocity dispersion, and the positively chirped single photons laser pulse is realized using attenuator 2 (ATT2) in our scheme. The other laser pulse is negatively chirped pulse after a broadband FBG1.

The positively chirped single photons laser pulse and the negatively chirped classical laser pulse are combined by a 1550 nm/1550 nm single mode beam splitter (SBS (50:50)) and coupled into the z-cut PPLN waveguide chip by the fiber pigtail. Two polarization controllers (PC) and two 200:1 single mode polarization beam splitter (SPBS) are used for adjusting the positively chirped single photons laser pulse and negatively chirped classical laser pulse to the TM mode, that it supports Type-0 (*ee* → *e*) phase matching in our experiment for the PPLN waveguide chip. A stable temperature controller is used to keep the PPLN waveguide chip’s temperature to maintain the phase-matching of the SFG process. The spectrally narrowed single photon pulse of higher frequency is generated in a PPLN waveguide chip after interference filter (IF), with a nominal bandwidth of 20 nm (FWHM) centred around 780 nm (loss is about 1.2 dB), and coupled to an optical-fiber-coupled spectrometer. Finally, the SFG photons are detected by a Silicon APD (SAPD), whose detection efficiency is up to 60% at 775 nm and dark count rate is 26 cps. In our experiment, a superconducting single photon detector (SSPD) is used to calibrate and monitor the counts of the positively chirped single photons, whose detection efficiency is up to 10% at 1551 nm and dark count rate is 600 cps. Using the IF, any residue of the positively or negatively chirped light has to be filtered out from the SFG photons by a factor of 10^−18^.

The 5.2 cm long reverse-proton-exchange PPLN waveguide chip is quasi–phase matched to perform the SFG process 1551 nm + 1551 nm → 775.5 nm. It is poled with a quasi-phase-matching (QPM) period of 19.6 μm, which incorporated single mode filters designed to match the single mode size of SMF-28 optical fiber and is optical fiber pigtailed with low coupling losses of about 0.7 dB. The PPLN waveguide chip has a total fiber-to-output-facet throughput of approximately −1.5 dB for the 1550-nm telecommunications band.

## Experimental Results and Discussion

**Figure 2:**
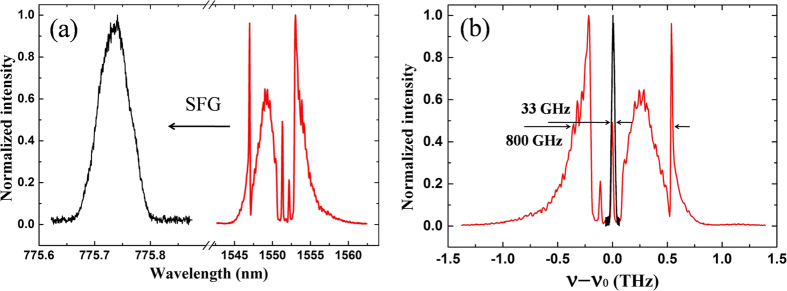
The positively chirped pulse spectrum and up-converted laser pulse spectrum (**a**) and relative frequency (**b**). The initial bandwidth of the positively chirped laser pulse is 800 GHz centred at 1551.54 nm (shown in red). Once the quadratic phase is applied and the two laser pulses are up-converted, the created laser pulse bandwidth reduces to 33 ± 1 GHz centred at 775.77 nm (shown in black). The spectra are given by normalized intensities and, for the up-converted case, correspond to the average of ten consecutive scans of 15 min acquisition time.

We first measure the spectrum of the positively chirped laser pulse by using an optical-fiber-coupled spectrometer and find a width 800 ± 20 GHz FWHM centred around 1551.54 nm. The positively chirped laser pulse is then sent into an optical fiber and superposed with the negatively chirped classical laser pulse (790 ± 20 GHz, centred around 1551.54 nm) in the PPLN waveguide chip for SFG. Here the FWHM bandwidths of both positively and negatively chirped photons are smaller than the spectral acceptance of the waveguide (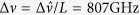). Thus, the full FWHM bandwidths of positively and negatively chirped photons are used in the waveguide, as expected. The up-converted laser pulse generated by the SFG process, after IF, is coupled into a single-mode optical fiber and coupled into the spectrometer. As shown in Fig. 2(a), we observe significant bandwidth compression of the positively chirped laser pulse. The measured bandwidth of the created laser pulse is Δ*ν*_*M*_ = 33 ± 1 GHz (FWHM), centred at 775.77 nm, where the relative time delay Δ*t* = 0. Taking the resolution of our spectrometer into account, Δ*ν*_*R*_ = 30 ± 1 GHz (FWHM), the actual bandwidth of the up-converted photons after deconvolution is 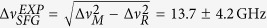 GHz (FWHM). This result agrees closely with theory, 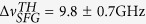 GHz (FWHM) from equation (1), using the expected chirp parameter *A* = (−2.52 ± 0.01) ∗ 10^8^ *fs*^2^ given by the geometry of our FBG. Therefore, a spectral compression ratio of 58:1 is realized in the positively chirped laser pulse frequency bandwidth (Fig. 2(b)).

**Figure 3:**
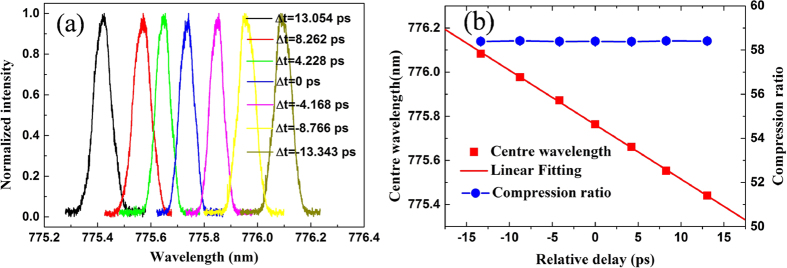
The SFG spectrum of the created laser pulse (**a**), central wavelength and compression ratio of the output pulses versus the optical relative delay (**b**). The central wavelength of the up-converted laser pulse is tuned by controlling the relative delay between the input pulses at the PPLN waveguide chip. Error bars are smaller than the data points.

The centre wavelength of the narrowband up-converted laser pulse can be tuned by adjusting the relative delay Δ*t* between the negatively and positively chirped laser pulses. The SFG spectrum of the created laser pulse could be given by a function of the delay, with the fitted centre wavelengths shown in Fig. 3. The experimental results show that the wavelength depends linearly on the delay, as expected. The linear fit gives a slope of −0.0247 ± 0.001 nm/ps. In terms of the slope data, we obtain the negatively chirp parameter of *A* = (−2.55 ± 0.01) ∗ 10^8^ *fs*^2^, in good agreement with the chirp parameter *A* = (−2.52 ± 0.01) ∗ 10^8^ *fs*^2^ of the FBG1. It is also found that the spectral compression ratio independent of the optical relative delay Δ*t*, which agrees closely with the theoretical result from the above eq. (1).

In our experiment, it is verified that any up-conversion single photons detected by the SAPD is the result of the SFG process, and not a second harmonic generation (SHG) of the positively or negatively chirped photons. Moreover, any residue of the positively or negatively chirped photons has to be filtered out from SFG photons by a factor of 10^−18^. When the number of photons per pulse of positively chirped light is attenuated to single-photon level, the detected SHG counts from the positively chirped photons drop to its dark counts (3.5 Hz). When the input energy of the negatively chirped laser pulse is less than 0.6 nJ, the SHG photons are also equal to the dark counts. The SFG efficiency is then given by *η*_*SFG*_ = *P*_*SFG*_/*βN*, where *P*_*SFG*_ represents the photons per second of SFG, *β* denotes the laser repetition rate, and *N* is average of photons per second of chirped laser pulse.

**Figure 4:**
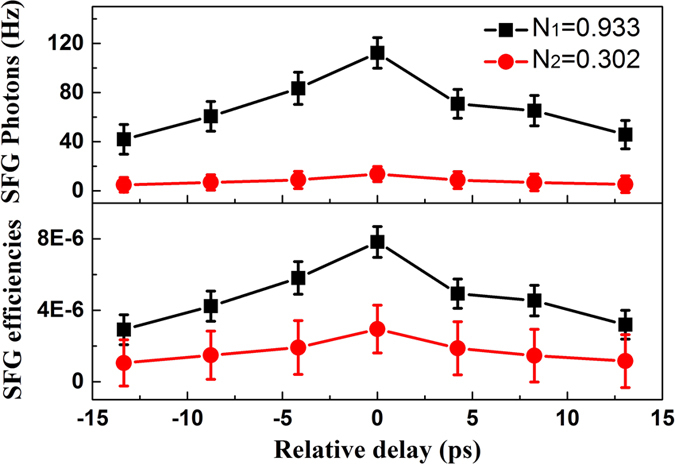
SFG photons (top) and SFG efficiencies (bottom). The dark counts (3.5 Hz) are subtracted. The SFG photons, SFG efficiencies and error bars are accounted and the abscissa is a variable optical relative delay between the negatively and positively chirped laser pulses at the PPLN waveguide chip.

As shown in Fig. 4, the energy of the negatively chirped laser pulse that is incident upon the PPLN waveguide chip is 0.6 nJ. By controlling the ATT2, the average of photons per second (*N*_1_ = 0.933 and *N*_2_ = 0.302) of positively chirped laser pulse can be obtained. SFG photons and SFG efficiencies of different situations are obtained by adjusting the relative delay Δ*t* (like Fig. 3(b)). At the same time, it is found that the overall conversion efficiency of SFG varies with the relative delay Δ*t*. When the relative time delay Δ*t* = 0, the maximum SFG efficiency is 7.82 × 10^−6^ with the average of photons of positively chirped laser pulse (0.933). Here the total losses have been considered, such as the coupling loss of 0.7 dB, total fiber-to-output-facet loss of 1.5 dB, reflection loss of 1.2 dB, and detection efficiency of 60% (see Fig. 4).

The lower SFG signal for single photons required longer times than for the intense photons states (to reduce the effects of drift in experimental parameters all the data in Fig. 4 are taken within a day). The results show that the average of photons of the positively chirped pulse are more, the efficiency of SFG is higher (see Fig. 4).

**Figure 5:**
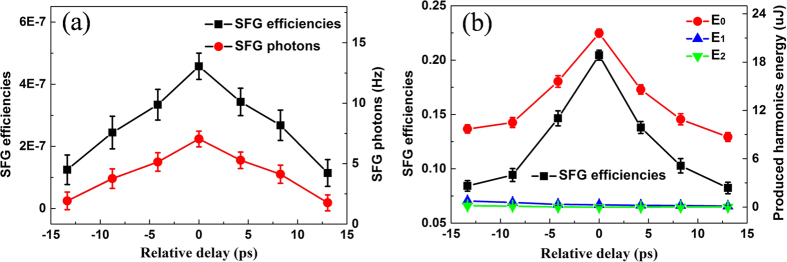
SFG photons and SFG efficiencies (**a**), SFG efficiencies and the energy of produced harmonics (**b**). The SFG efficiencies, SFG photons, the energy of produced harmonics, and error bars of them are accounted and the abscissa is a variable optical relative delay between the negatively and positively chirped laser pulses at the PPLN waveguide chip. The dark counts (3.5 Hz) are subtracted.

It is found that the SFG efficiency decreases with reducing the negatively chirped photons, and the SFG efficiency also increases with increasing input energy of the positively and negatively chirped light. Next, we measure the SFG efficiency in two ways: one, by attenuating the negatively chirped photons with the ATT1 and ATT2; the other by increasing the energy of positively and negatively chirped light with the ATT1 and ATT2. Figure 5 depicts the results of these two measurements.

As shown in Fig. 5(a), when the negatively chirped photons (ten photons per pulse) and positively chirped single photons laser pulse (0.933 photons per pulse) are simultaneously sent to the waveguide, the maximum SFG efficiency of 4.58 × 10^−7^ is realized, where the relative time delay Δ*t* = 0. As shown in Fig. 5(b), by adjusting the ATT1 and ATT2, we control the input energy of positively and negatively chirped light is 203.1 μJ and 202.8 μJ, respectively. The energy of produced harmonics *E*_*i*_(*i* = 0, 1, 2) is measured, where *E*_0_ is the total energy of SFG and SHG of positively and negatively chirped light, *E*_1_ is the energy of SHG of positively chirped light, *E*_2_ is the energy of SHG of negatively chirped light. When the relative time delay Δ*t* = 0, the energy of produced harmonics is *E*_0_ = 21.62 *μJ*, which is obtained from SHG of positively chirped light (*E*_1_ = 0.28 *μJ*), SHG of negatively chirped light (*E*_2_ = 0.01 *μJ*), and SFG (*E*_*SFG*_ = *E*_0_ − *E*_1_ − *E*_2_ = 21.33 *μJ*). In the case, the maximum SFG efficiency of 20% is obtained, where the total losses have been taken into account. We note that the rate of generated photons by SFG is 73 times of the rate of photons generated by the SHG of these two independent sources.

**Figure 6:**
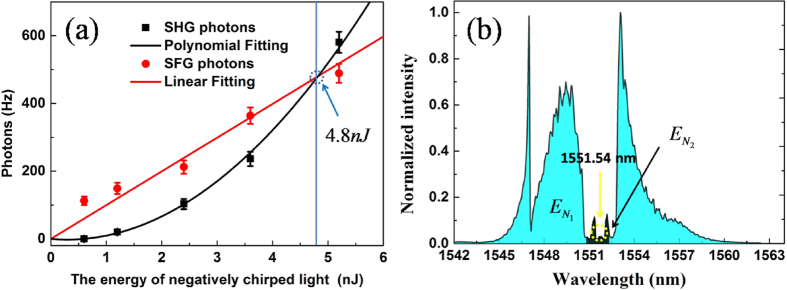
SHG photons and SFG photons (**a**), the negatively chirped pulse spectrum (**b**). The dark counts (3.5 Hz) are subtracted, and the error bars are accounted.

In our scheme, although both negatively and positively chirped laser pulses are at the same center wavelength, the energy of SHG from each is kept below the energy of SFG (see Fig. 5(b)). At the same time, the SHG photons from the two beams are also lower than the SFG photons when the bandwidth of single-photon level positively chirped laser pulse is compressed (see Figs 4 and 5(a)). We can also confirm that by adjusting the energy of negatively chirped light, considering a positively chirped single-photon level laser pulse (0.933 photons per pulse). Figure 6 depicts the results of experimental and theoretical SFG and SHG.

In the experiment, when the input energy of the negatively chirped laser pulse is more than 0.6 nJ, the up-converted single photons consists of SHG photons of negatively chirped laser pulse, and SFG photons of the negatively chirped laser pulse and positively chirped single photons laser pulse. Although up-converted single photons cannot be separately distinguished, the SFG photons *P*_*SFG*_ can be measured. In the experiment, we first couple the negatively chirped laser pulse into the PPLN waveguide chip alone, and we measure the SHG photons *P*_1_ of the negatively chirped laser pulse. If we simultaneously send the negatively chirped laser pulse and positively chirped single photons laser pulse together to the PPLN waveguide chip, we obtain the photons *P*_0_ of SFG and SHG. The photons generation of SFG are calculated according to the equation *P*_*SFG*_ = *P*_0_ − *P*_1_. When the relative time delay Δ*t* = 0, the SHG and SFG photons of different situations are obtained by adjusting the energy of negatively chirped light (see Fig. 6(a)). The measured results show that SHG photons are lower than the SFG photons when the energy of negatively chirped light is less than 4.8 nJ. When *P*_*SFG*_ = *P*_1_, the SFG efficiency of 3.12 × 10^−5^ is obtained. If the energy of negatively chirped light is more than 4.8 nJ, SHG photons will be more than the SFG photons.

Next, we carry out the theoretical analysis for SHG and SFG photons. As shown in Fig. 6(b), the spectrum of the negatively chirped laser pulse is measured. It is shown that the intensity of negatively chirped laser pulse at 1551.54 nm is very close to zero. When the negatively chirped light is coupled into the waveguide alone, the SHG photons are very low. Here we assume that the energy of negatively chirped light which can be converted into SHG photons is 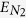
(Black area), thus, the SHG photons 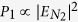. However, when the negatively and positively chirped photons are simultaneously sent together to the waveguide, photons in all spectrum of the negatively chirped light can be used to perform the SFG process. The full energy of negatively chirped light is 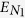, as shown in Fig. 6(b). Thus, the SFG photons 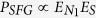, where *E*_*S*_ is the energy of positively chirped single-photon level pulse. When *P*_*SFG*_/*P*_1_ ≥ 1, SFG photons are more than the SHG photons. In the case, we obtain


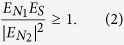


In terms of 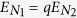, one can obtain 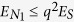, where *q* is proportional coefficient. In the experiment, one has *E*_*S*_ = 7.3 × 10^−3^ *nJ* and *q* = 26.8, thus, 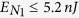 is realized. This result agrees closely with experiment, as shown in Fig. 6(a).

In order to reduce the SHG photons to the dark counts (3.5 Hz), the energy of negatively chirped light must be very low. Thus, the SFG efficiency is limited. If the wavelengths centred at 1551.54 nm of negatively chirped light are filtered out using a filter, the SFG efficiency may be increased a lot. Furthermore, our results may provide potential application in standard decoy-state quantum key distribution. By considering a fiber attenuation of 0.2 dB/km, the dark counts of 3.5 Hz, the coupling loss of 0.7 dB, total fiber-to-output-facet loss of 1.5 dB, reflection loss of 1.2 dB, detection efficiency of 60%, and sources with a 59.98 MHz repetition rate, the SFG efficiency of 7.82 × 10^−6^ will achieve a rate of about 8 bits/hour on a distance of 20 km.

## Conclusions

In conclusion, we have experimentally demonstrated that the spectrum of single photons laser pulse was compressed by a factor of 58 in a PPLN waveguide chip. A positively chirped single photons laser pulse and a negatively chirped classical laser pulse by fiber Bragg gratings were used to produce a narrow bandwidth single photon pulse with new frequency through SFG process. The frequency and bandwidth of single photons at 1550 nm were simultaneously converted. In addition, the entire experiment is fiber-coupled. Our results have demonstrated the potentially application for PPLN waveguide chip as an integrated platform for spectrum compressing and frequency conversing in the telecom band, such as coherent photonic interfaces between quantum communication at 1550 nm and quantum memory in the near-visible window.

## Additional Information

**How to cite this article**: Li, Y. *et al*. Spectral compression of single-photon-level laser pulse. *Sci. Rep.* **7**, 43494; doi: 10.1038/srep43494 (2017).

**Publisher's note:** Springer Nature remains neutral with regard to jurisdictional claims in published maps and institutional affiliations.

## References

[b1] Comandar L. C. . Quantum key distribution without detector vulnerabilities using optically seeded lasers. Nature Photon. 10, 312–315 (2016).

[b2] Sasaki T., Yamamoto Y. & Koashi M. Practical quantum key distribution protocol without monitoring signal disturbance. Nature 509, 475–478 (2014). 24848060 10.1038/nature13303

[b3] Guan J. Y. . Experimental passive round-robin differential phase-shift quantum key distribution. Phys. Rev. Lett. 114, 180502 (2015). 26000991 10.1103/PhysRevLett.114.180502

[b4] Napolitano M. . Interaction-based quantum metrology showing scaling beyond the Heisenberg limit. Nature 471, 486–489 (2011). 21430776 10.1038/nature09778

[b5] Miller J. & Miyake A. Resource quality of a symmetry-protected topologically ordered phase for quantum computation. Phys. Rev. Lett. 114, 120506 (2015). 25860730 10.1103/PhysRevLett.114.120506

[b6] Gisin N. . Quantum cryptography. Rev. Mod. Phys. 74, 145–195 (2002).

[b7] Eisaman M. D. . Invited review article: single-photon sources and detectors. Rev. Sci. Instrum. 82, 071101 (2011). 21806165 10.1063/1.3610677

[b8] Zhang L. . Experimental investigation on line width compression of stimulated Brillouin scattering in water. Appl. Phys. Lett. 98, 221106 (2011).

[b9] Rusu M. & Okhotnikov O. G. All-fiber picosecond laser source based on nonlinear spectral compression. Appl. Phys. Lett. 89, 091118 (2006).

[b10] Matsuda Y. . Spectral compression of ultrafast acoustic transients in thin films for enhanced detectability. Appl. Phys. Lett. 79, 2288–2290 (2001).

[b11] Khaydarov J. D. V. . 20-fold pulse compression in a synchronously pumped optical parametric oscillator. Appl. Phys. Lett. 65, 1614–1616 (1994). 10.1364/ol.19.000831 19844460

[b12] Raoult F. . Efficient generation of narrow-bandwidth picosecond pulses by frequency doubling of femtosecond chirped pulses. Opt. Lett. 23, 1117–1119 (1998). 18087446 10.1364/ol.23.001117

[b13] Nejbauer M. . Spectral compression of femtosecond pulses using chirped volume Bragg gratings. Opt. Lett. 41, 2394–2397 (2016). 27244372 10.1364/OL.41.002394

[b14] Donohue J. M., Mazurek M. D. & Resch K. J. Theory of high-efficiency sum-frequency generation for single-photon waveform conversion. Phys. Rev. A 91, 033809 (2015).

[b15] Pontecorvo E. . Spectrally tailored narrowband pulses for femtosecond stimulated Raman spectroscopy in the range 330–750 nm. Opt. Express 21, 6866–6872 (2013). 23546068 10.1364/OE.21.006866

[b16] Hoffman D. P. . Optimally shaped narrowband picosecond pulses for femtosecond stimulated Raman spectroscopy. Opt. Express 21, 21685–21692 (2013). 24104042 10.1364/OE.21.021685

[b17] Chao W. T. . Adiabatic pulse propagation in a dispersion-increasing fiber for spectral compression exceeding the fiber dispersion ratio limitation. Opt. Lett. 39, 853–856 (2014). 24562224 10.1364/OL.39.000853

[b18] Huang Z. Y. . Few-cycle laser pulses generation with frequency tuning in a molecular gas-filled hollow-core fiber. Opt. Express 23, 17711–17719 (2015). 26191833 10.1364/OE.23.017711

[b19] Mandeng L. M. . Spectral compression in the supercontinuum generation through the higher-order nonlinear Schrödinger equation with non-Kerr terms using subnanojoule femtosecond pulses. J. Opt. Soc. Am. B 30, 2555–2559 (2013).

[b20] Bao C., Xiao X. & Yang C. Spectral compression of a dispersion-managed mode-locked Tm: fiber laser at 1.9 μm. IEEE Photon. Technol. Lett. 28, 497–500 (2015).

[b21] Finot C. & Boscolo S. Design rules for nonlinear spectral compression in optical fibers. J. Opt. Soc. Am. B 33, 760–767 (2016).

[b22] Marceau C. . Femtosecond laser pulse compression using angle of incidence optimization of chirped mirrors. Laser Phys. Lett., 11, 065302 (2014).

[b23] Lassonde P. . High energy femtosecond pulse compression. Laser Phys. Lett., 13, 075401 (2016).

[b24] Zheng Y. L. . Optically induced transparency in a micro-cavity. Light: Science & Applications 5, 16072 (2016). 10.1038/lsa.2016.72 PMC6059932 30167162

[b25] Brecht B. . Demonstration of coherent time-frequency Schmidt mode selection using dispersion-engineered frequency conversion. Phys. Rev. A 90, 030302(R) (2014).

[b26] Rakher M. T. . Quantum transduction of telecommunications-band single photons from a quantum dot by frequency upconversion. Nature Photon. 4, 786–791 (2010).

[b27] Tanzilli S. . A photonic quantum information interface. Nature 437, 116–120 (2005). 16136138 10.1038/nature04009

[b28] Zhou Z.-Y. . Generation of light with controllable spatial patterns via the sum frequency in quasi-phase matching crystals. Sci. Rep. 4, 5650 (2014). 25007780 10.1038/srep05650 PMC4090625

[b29] Steinlechner F. . Frequency conversion of structured light. Sci. Rep. 6, 21390 (2016). 26875448 10.1038/srep21390 PMC4753436

[b30] Aktas D. . Entanglement distribution over 150 km in wavelength division multiplexed channels for quantum cryptography. Laser & Photon. Rev. 10, 451–457 (2016).

[b31] Sun Q. C. . Experimental passive decoy-state quantum key distribution. Laser Phys. Lett., 11, 085202 (2014).

[b32] Liu H. G. . Nonlinear Raman-Nath second harmonic generation with structured fundamental wave. Optics Express. 24, 15666–15671 (2016). 27410839 10.1364/OE.24.015666

[b33] Kielpinski D., Corney J. F. & Wiseman H. M. Quantum optical waveform conversion. Phys. Rev. Lett. 106, 130501 (2011). 21517362 10.1103/PhysRevLett.106.130501

[b34] Lavoie J. . Spectral compression of single photons. Nature Photon. 7, 363–366 (2013).

[b35] Rakher M. T. . Simultaneous wavelength translation and amplitude modulation of single photons from a quantum dot. Phys. Rev. Lett. 107, 083602 (2011). 21929167 10.1103/PhysRevLett.107.083602

[b36] Fisher K. A. G. . Frequency and bandwidth conversion of single photons in a room-temperature diamond quantum memory. Nature Commun. 7, 11200 (2016). 27045988 10.1038/ncomms11200 PMC4822040

[b37] Reim K. F. . Towards high-speed optical quantum memories. Nature Photon. 4, 218–221 (2010).

[b38] Reim K. F. . Single-photon-level quantum memory at room temperature. Phys. Rev. Lett. 107, 053603 (2011). 21867069 10.1103/PhysRevLett.107.053603

